# Clinicopathological Characteristics of Mucinous Breast Cancer: A Retrospective Analysis of a 10-Year Study

**DOI:** 10.1371/journal.pone.0155132

**Published:** 2016-05-27

**Authors:** Lei Lei, Xingfei Yu, Bo Chen, Zhanhong Chen, Xiaojia Wang

**Affiliations:** 1 Department of Medical Oncology, Zhejiang Cancer Hospital, Hangzhou, Zhejiang Province, P. R. China; 2 Department of Breast Tumor Surgery, Zhejiang Cancer Hospital, Hangzhou, Zhejiang Province, P. R. China; 3 Department of Pathology, Zhejiang Cancer Hospital, Hangzhou, Zhejiang Province, P. R. China; INRS, CANADA

## Abstract

**Background:**

Mucinous breast carcinoma (MC) is a special type of breast cancer that presents with a large amount of extracellular mucin. MC comprises approximately 4% of all invasive breast cancers. This type of tumor has a better prognosis and higher incidence in peri- and post-menopausal patients. Pathologically, there are two main subtypes of MC: pure and mixed. In this study, we describe 10 years of experience with MC at the Zhejiang Cancer Hospital in China, specifically, clinical data, histological findings and immunohistochemical features.

**Methods:**

We identified MC patients who were diagnosed as operable and completed clinical treatment from January 2001 to January 2011. The clinicopathological data included the age at diagnosis, tumor size, TNM stage, presence and number of lymph node (LN) metastases, estrogen receptor (ER), progesterone receptor (PR) and human epidermal growth factor receptor-2 (HER2) status and p53 expression. If the tumor was defined as mixed mucinous carcinoma (MMC), IHC was performed on a non-mucinous part, such as invasive ductal and lobular cancer. We evaluated the clinical characteristics of all MC patients using chi-square, one-way ANOVA and LSD tests. We also studied the correlations between all of the clinical parameters and LN metastasis in a binary logistic regression analysis. We used ten consecutive years of data that were collected at Zhejiang Cancer Hospital.

**Results:**

We identified 48 cases of pure mucinous carcinoma (PMC) and 77 cases of MMC. The 48 PMC cases consisted of 38 PMC-A and 10 PMC-B subtypes. The MMCs were divided into two groups, those with partial mixed mucinous breast carcinoma (pMMC, 58 cases) and those with main mixed mucinous breast carcinoma (mMMC, 19 cases). pMMC was defined by tumors with less than 50% mucinous components, while mMMC was defined by tumors where the mucinous component accounted for 50% to 90% of the tumor. No significant differences in the clinicopathological characteristics were noted between the patients with PMC-A and those with PMC-B. The tumor size was larger in the mMMC than PMC cases (44.84 mm vs. 30.06 mm, p = 0.021). The number of positive LN metastases was greater in pMMC than PMC patients (p = 0.024). The clinical stages were significantly different among the three groups, with the pMMC group having more stage III-IV patients than the other two groups (p = 0.005). The incidence of LN metastasis was also higher in the pMMC cases (pMMC vs. mMMC and PMC, 50% vs. 31.58% and 18.75%, p = 0.003). The PMC patients had much lower p53 expression than the other two groups (PMC vs. pMMC and mMMC, 27.08% vs. 55.17% and 57.89%, p = 0.007). The tumor size (>30mm), p53 expression and less proportion of the mucinous component are associated with risk of LN metastasis.

**Conclusion:**

Based on the results of this study, we conclude that the tumor size, status of LN metastasis, clinical stage, and p53 mutation rate may differ between MMC and PMC patients. The tumor size (>30mm), p53 mutation and less proportion of the mucinous component should be considered risk factors of LN metastasis in MC patients.

## Introduction

Mucinous breast carcinoma (MC) is a special type of breast cancer that presents with a large amount of extracellular mucin. MC comprises approximately 4% (range: 1% to 7%) of all invasive breast cancers [1–3]. MC has a better prognosis (90% survival at 10 years) and a higher incidence in peri- and post-menopausal patients [4]. Pathologically, MC is divided into two main subtypes, pure and mixed [5]. The distinction between these subtypes is based on the quantification of cellularity. The mucinous component varies from 30% to over 90% of the tumor [6]. Currently, there is no established percentage to make a positive diagnosis of mucinous carcinoma. However, most pathologists agree that a diagnosis of pure mucinous carcinoma (PMC) should be reserved for tumors with at least 90% mucinous components [7]. The pure type almost exclusively consists of tumor tissue with extracellular mucin production, while the mixed subtype also contains other *in situ* or invasive components without mucin.

PMC represents approximately 2% of all malignant breast carcinomas [1]. This type of cancer is most commonly diagnosed in women aged 55–67 and above [6,8–17]. The characteristic feature of this type of cancer is that it can be diagnosed at relatively early stages of the disease. Stage T1–2 tumors are diagnosed in 75%–97% of patients, and a lack of metastases to lymph nodes is observed in 62%-88% of patients [6,11,18–23]. Usually, PMC is ER and PR positive and AR negative [8]. PMC may be subtyped into a hypocellular variant (PMC-A), showing a tubular, cribriform, cord-like, micropapillary or papillary growth pattern, and a hypercellular variant (PMC-B), growing in solid nests [24]. Conventionally, PMC exhibits a metastasis rate of less than 15% [25] and has a better prognosis than invasive breast carcinoma of no special type [2,7,26].

Mixed mucinous carcinoma (MMC) is mainly associated with lobular or ductal neoplasia (*in situ* or invasive), and some tumors have neuroendocrine differentiation [3]. However, a specific percentage has not been clearly established for MMC diagnosis. Due to the distinct clinicopathological parameters of PMC and MMC, there may be a prognostic difference between the two groups. The 10-year survival rate of the pure type (90.4%) was better (p<0.001) than that of the mixed type (66.0%) [27].

The purpose of this paper is to report on 10 years of experience with MC at Zhejiang Cancer Hospital in China, specifically clinical data, histological findings and immunohistochemical features.

### Ethics statement

The Zhejiang Cancer Hospital Ethic Institution Office approved this study. This retrospective study poses no potential risk to the subjects, and the subjects’ personal privacy was protected. This study strictly conforms to the principles outlined in the Declaration of Helsinki.

## Materials and Methods

**Fig 1:**
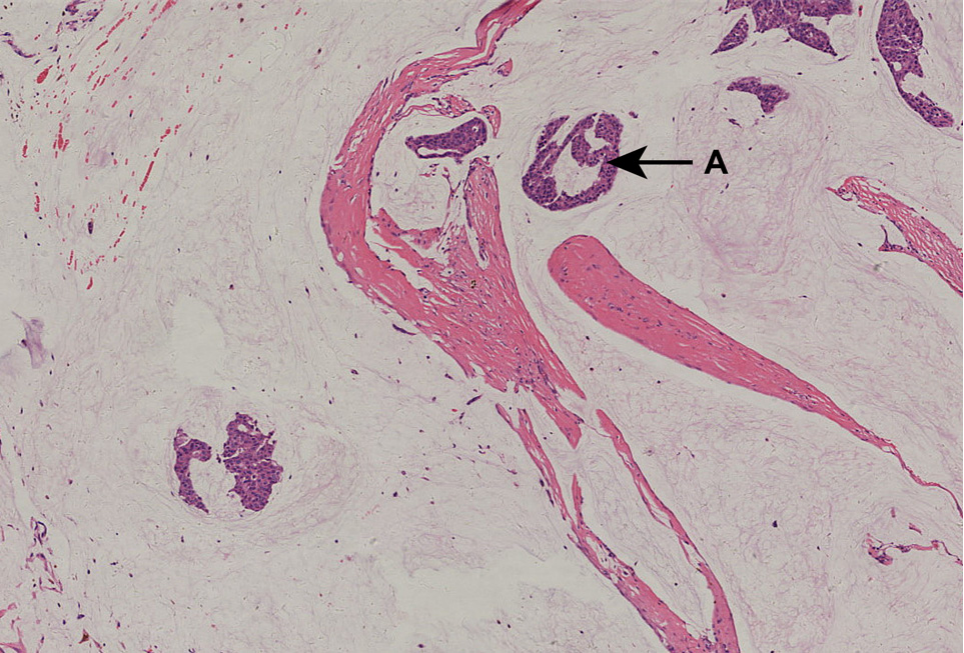
A 51-year-old woman with stage IIA pT2N0M0 PMC. Pathological diagnosis was made using H&E staining, which showed PMC—hypocellular variant, sparse nests or individual cells floating in a large amount of mucin, segmented by fibers. ER (3+), PR (3+), CerbB-2 (-). All images had an original magnification of ×40 and were H&E stained unless otherwise indicated. A (arrow) shows mucinous breast carcinoma. Abbreviations: H&E, hematoxylin and eosin; PMC, pure mucinous breast carcinoma; staging was performed according to the seventh edition of the TNM Classification for Breast Cancer.

**Fig 2:**
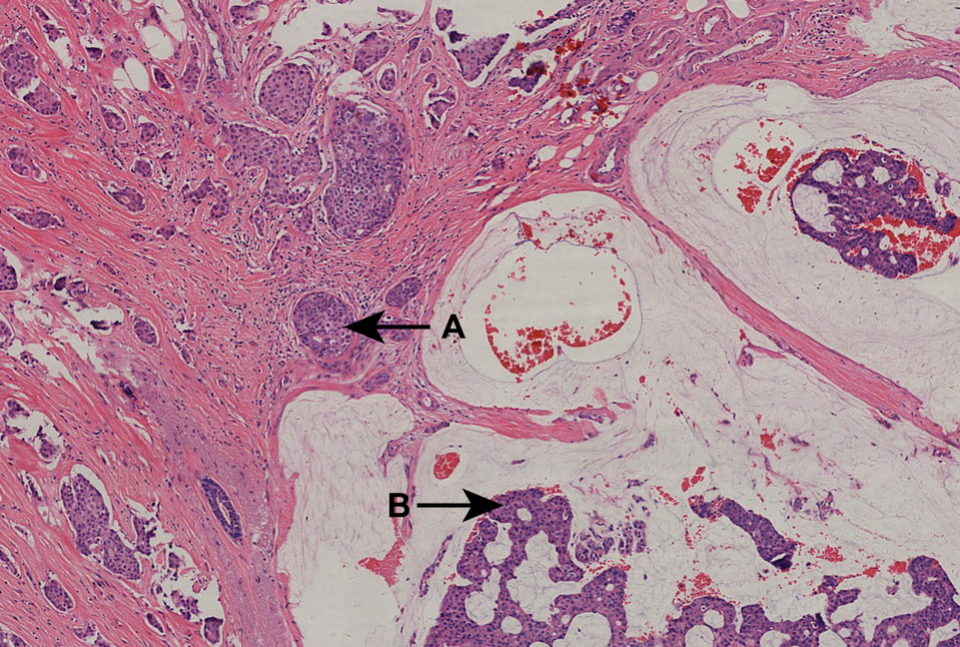
A 53-year-old woman with stage IIB pT3N0M0 MMC. Pathological diagnosis was determined by H&E staining and showed MMC—mucinous carcinoma mixed with invasive ductal breast carcinoma. ER (3+), PR (-), CerbB-2 (+). All images had an original magnification of ×40 and were H&E stained unless otherwise indicated. A (arrow) indicates invasive ductal breast carcinoma. B (arrow) indicates mucinous breast carcinoma. Abbreviations: H&E, hematoxylin and eosin; MMC, mixed mucinous breast carcinoma; staging according to the seventh edition of the TNM classification for breast cancer.

Patients diagnosed with PMC (Fig 1) and MMC (Fig 2) according to the WHO Classification of Tumours of the Breast 2012 [8] between 2001 and 2011 at Zhejiang Cancer Hospital in China were retrospectively included in this study. The clinicopathological data evaluated included age at diagnosis, tumor size (mm), TNM stage (AJCC 7^th^ ed.), p53 expression, status and number of metastatic lymph nodes (LNs), and expression of estrogen receptor (ER), progesterone receptor (PR), and human epidermal growth factor receptor-2 (HER2). The PMC patients were subtyped into PMC-A and PMC-B groups for comparison purposes. All immunohistochemical materials (i.e., HE slices, immunohistochemical staining slices and paraffin-embedded slices) were reassessed, and the findings were confirmed by professional pathologists. If the tumor was defined as MMC, IHC was performed on a non-mucinous part, such as invasive ductal and lobular cancer. ER, PR and p53 expression were measured using semi-quantitative cell nucleus scores. Cases that were 3+ by IHC or + by fluorescence *in situ* hybridization (FISH) were defined as HER2-positive. Cases that were IHC 1+/or FISH- were considered negative for HER2 expression. Cases with IHC 2+ were further tested by FISH to confirm HER2 status. Data collection and processing were performed using SPSS (v22.0). We used χ2 tests (and Fisher’s exact test, if necessary) to analyze qualitative data. Comparisons of the age at diagnosis, tumor size and number of positive LNs among the three subgroups of MC were tested by one-way ANOVA and LSD tests. Logistic regression was used in multivariate analyses to identify risk factors impacting LN metastasis. The age at diagnosis (>40y), tumor size (>30mm), ER/PR/HER-2/P53 status and subgroups of MC were included as potential risk factors. The significance level was defined as p<0.05.

## Results

In total, 48 patients were diagnosed with PMC, and 77 patients were diagnosed with MMC at the Zhejiang Cancer Hospital. All MC patients represented 2.4% of all (5,297) patients treated for breast cancer during this period. The 48 PMC cases consisted of 38 PMC-A and 10 PMC-B cases. No significant differences in the clinicopathological characteristics were noted between the patients with PMC-A and those with PMC-B (Table 1). The MMC patients were divided into two groups: those with partial mixed mucinous breast carcinoma (pMMC, 58 cases) and those with main mixed mucinous breast carcinoma (mMMC, 19 cases). The pMMC was defined as tumors with <50% mucinous components, and mMMC was defined as tumors where the mucinous component accounted for 50% to 90% of the tumor. The additional invasive components in MMC included infiltrative ductal breast cancer (51 cases), lobular breast cancer (two cases) and other types of breast cancer (five cases).

**Table 1:** Correlations between subtype and clinicopathological parameters in 48 PMC patients^$^.

Parameters		PMC(N = 48)		Significance
		PPMC-A(N = 38)	PPMC-B(N = 10)	
Age (years, mean±SD)		50.58±13.29	57.80±12.37	F = 2.400, p = 0.128
Tumor size (mm, mean±SD)		30.13±22.51	29.80±13.25	F = 0.002, p = 0.965
Number of positive LN (mean±SD)		0.87±2.76	0.10±0.32	F = 0.759, p = 0.388
LN status	Negative	30	9	χ^2^ = 0.635, p = 0.426
	Positive	8	1	
Stage	I	11	3	χ^2^ = 0.850, p = 0.838
	II	24	7	
	III	2	0	
	IV	1	0	
ER	Negative	5	2	χ^2^ = 0.298, p = 0.585
	Positive	33	8	
PR	Negative	9	4	χ^2^ = 1.067, p = 0.302
	Positive	29	6	
HER2	Negative	36	9	χ^2^ = 0.303, p = 0.582
	Positive	2	1	
p53	Negative	26	9	χ^2^ = 1.867, p = 0.172
	Positive	12	1	

^**$**^ PMC: pure mucinous breast carcinoma; PMC is subtyped into hypocellular PMC (PMC-A) and hypercellular PMC (PMC-B).

The mean ages of all groups were similar (pMMC vs. mMMC vs. PMC, 50.66 vs. 52.08 vs. 52.26 years, respectively, p>0.05). The tumor size was larger in the mMMC than in the PMC group (44.84 mm vs. 30.06 mm, p = 0.021). The clinical stages were significantly different among the three groups (p = 0.005). The pMMC group had more stage III-IV patients than the other groups. LN metastasis was also more common in the pMMC patients (pMMC vs. mMMC and PMC, 50% vs. 31.58% and 18.75%, respectively, p = 0.003). The number of positive LN metastases was higher in pMMC than PMC patients (p = 0.024). ER positivity was found in 41 (85.41%) of the PMC cases and 66 (85.71%) of the MMC cases. PR positivity was found in 35 (72.92%) of the PMC cases and 51 (66.23%) of the MMC cases. HER-2 positivity was found in three (6.25%) of the PMC cases and 11 (14.29%) of the MMC cases. The rate of positivity for ER, PR and HER-2 was similar among the three groups (p>0.05). The PMC patients had much lower p53 expression than the other two groups (PMC vs. pMMC and mMMC, 27.08% vs. 55.17% and 57.89%, respectively, p = 0.007) (Table 2). The tumor size (>30mm), p53 expression and subgrouping of MC according to the proportion of the mucinous component were associated with LN metastasis (Table 3).

**Table 2:** Clinical, histopathological and immunohistochemical findings of PMC in comparison with pMMC and mMMC^¶^.

Parameters		pMMC(N = 58)	mMMC (N = 19)	PMC (N = 48)	Significance
Age (years, mean±SD)		50.66±11.60	52.08±13.31	52.26±11.99	F = 0.225, p = 0.799
Tumor size (mm, mean±SD)		37.84±21.70	44.84±32.79	30.06±20.80	F* = 3.094, p* = 0.049
Number of positive LN (mean±SD)		2.90±4.95	0.71±2.48	3.26±8.36	F^#^ = 3.222, p^#^ = 0.043
LN status	Negative	29	13	39	χ^2^ = 11.373, p = 0.003
	Positive	29	6	9	
Stage	I	12	3	14	χ^2^ = 18.773, p = 0.005
	II	28	13	31	
	III	17	1	2	
	IV	1	2	1	
ER	Negative	10	1	7	χ^2^ = 1.668, p = 0.434
	Positive	48	18	41	
PR	Negative	19	7	13	χ^2^ = 0.726, p = 0.695
	Positive	39	12	35	
HER2	Negative	48	18	45	χ^2^ = 3.984, p = 0.136
	Positive	10	1	3	
p53	Negative	26	8	35	χ^2^ = 9.933, p = 0.007
	Positive	32	11	13	

^¶^ PMC: pure mucinous breast carcinoma, mucinous component >90%; pMMC: partial mixed mucinous breast carcinoma, mucinous component <50%; mMMC: main mixed mucinous breast carcinoma, mucinous component 50%~90%.

* pMMC/mMMC, p = 0.259; pMMC/PMC, p = 0.090; mMMC /PMC, p = 0.021.

^#^ pMMC/mMMC, p = 0.778; pMMC/PMC, p = 0.024; mMMC/PMC, p = 0.057.

**Table 3:** Logistic regression analysis results of factors predicting LN metastasis.

Parameters	B	S.E.	Wald	df	Sig.	Exp(B)	95.0% C.I. for EXP(B)	
							Lower	Upper
Age(>40y)	-0.346	0.609	0.323	1	0.570	0.707	0.214	2.335
Tumor size(>30mm)	1.731	0.508	11.620	1	0.001	5.648	2.087	15.281
ER+	-0.079	0.697	0.013	1	0.910	0.924	0.236	3.623
PR+	-0.366	0.524	0.489	1	0.484	0.693	0.248	1.937
HER2+	1.084	0.761	2.027	1	0.154	2.956	0.665	13.140
P53+	1.268	0.450	7.959	1	0.005	3.554	1.473	8.578
Subgroups of MC^§^	-0.425	0.258	2.850	1	0.046	0.847	0.391	0.972
Constant	-2.911	1.514	3.697	1	0.055	0.054		

§ Subgroups of MC: pMMC, mMMC and PMC groups.

## Discussion

PMC represents approximately 2% of all breast cancers [8]. In comparison to infiltrating ductal carcinoma of the breast, PMC has better prognostic features. According to the literature, the 5-year and 10-year survival rates of infiltrating ductal carcinoma and PMC are 77%–92% and 75%–89% (overall), respectively, and 76%–91% and 74%–93% (disease-free), respectively [6,9,11,18–19,21,27–29]. Pathological characteristics are dominant in approximately 90%-100% of PMC cases. No significant difference in clinicopathological characteristics was noted in our study between the PMC-A and PMC-B subtypes. This result is consistent with those obtained in some previous studies [24]. Therefore, we compared the combination of PMC-A and PMC-B patients with other groups of MC. In the mixed form of MC, other invasive carcinoma components are also present.

Although an exact percentage necessary to diagnose mucinous carcinoma has not been clearly established, most pathologists agree that a diagnosis of PMC should be reserved for tumors with a mucinous component of at least 90%. It is important to clearly distinguish PMC from MMC, with the latter containing a mixture of mucinous and non-mucinous components, because the prognosis of pure mucinous carcinoma seems to be better than that of MMC [17]. In our study, MMC was sub-divided into two groups based on the mixed mucinous component. To the best of our knowledge, there is no definite cutoff in terms of the percentage for diagnosing partial or major mucinous components in MMC. However, based on our current data, MMC with a smaller mucinous component presented with worse pathological factors, such as LN metastasis, more advanced clinical stage and higher p53 expression. These results are consistent with the clinical and immunohistochemical findings of a recent study comparing PMC with MMC [21].

Cao et al. retrospectively compared the clinical and biological features between 2,202 patients with pure invasive ductal carcinoma and 309 patients with PMC treated from 1999 to 2010 in China. They found that PMC had favorable characteristics, including a smaller tumor size, lower rates of LN positivity, lower stage, higher expression of hormone receptors, and less HER2 overexpression[23]. In our study, the smaller tumor size, lower rate of LN metastasis and lower stage were consistent with their results. However, the ER/PR/HER2 status was not significantly different among the three groups. This may be explained by the fact that the diagnostic standards for pathological studies changed several times during the study period.

According to previous data, local and distant failures in patients with mucinous breast cancer are rare and occur in fewer than 6% of patients [6,11,22]. Diab et al. and Vo et al. claim that the decisive risk factor for the development of these failures is the presence of LN metastases [9,11]. The inclusion of tumor size in the staging system may not be a significant factor, because mucin comprises the majority of the tumor volume [30]. Furthermore, as was indicated by Vo et al., failures are less frequently noted in patients with MC in comparison with other histological types of breast cancer [11]. In our study, we found that LN metastases and clinical stage were related to the extent of the mucinous component in MC. A smaller mucinous component corresponded to more lymph node metastases and worse clinical staging.

Erhan et al. [21] noted that MMC also displayed higher p53 expression than PMC (30% vs. 8%; p<0.05). HER2 positive status was noted in 42% of the MMC cases, a ratio that was higher than that in PMC (4%; p<0.001). Our study supported the findings by Erhan et al. and also found that p53 expression was different among the three groups and corresponded to the size of the mucinous component.

However, some proliferation-related markers, such as Ki-67 and Topo II, were not regularly tested in our center until 2008. We chose 50% as a cutoff percentage for diagnosing partial or major mucinous components in MMC according to clinical practice. More studies are warranted to determine a precise definition of partial or major mucinous components in MMC.

## Conclusion

Based on the results from this study, we conclude that the tumor size, status of LN metastasis, clinical stage, and p53 mutation rate may differ between MMC and PMC patients. The tumor size (>30mm), p53 mutation and less proportion of the mucinous component should be considered risk factors of LN metastasis in MC patients.
